# Bear Maul Injury among Patients Presenting to the Department of Surgery in a Tertiary Care Centre: A Descriptive Cross-sectional Study

**DOI:** 10.31729/jnma.7907

**Published:** 2022-12-31

**Authors:** Suryaman Menyangbo, Gakul Bhatta, Poojan Kumar Rokaya, Rabin Basnet

**Affiliations:** 1 Department of General Surgery, Karnali Academy of Health Sciences, Chandannath, Jumla, Nepal; 2 Department of Orthopaedics, Karnali Academy of Health Sciences, Chandannath, Jumla, Nepal; 3 Department of Emergency, Bir Hospital, Kantipath, Kathmandu, Nepal

**Keywords:** *bears*, *injuries*, *lacerations*, *Nepal*, *prevalence*

## Abstract

**Introduction:**

Bears are robust and agile wild creatures that can potentially inflict injuries. Black bears *(Ursus thibetanus)* are an inhabitant of the mountainous part of Nepal. Encounters between humans and bears generally occur in the bear-prevalent areas of Nepal and the world inflicting complex categorical injuries. The aim of the study was to find the prevalence of bear maul injury among patients presenting to the Department of Surgery in a tertiary care centre.

**Methods:**

A descriptive cross-sectional study was done in the Department of Surgery in a tertiary care centre from 1 August 2017 to 1 September 2022. Data was collected from the records of the Department of Surgery after receiving ethical approval from the Institutional Review Committee (Reference number: 078/79/38). The collected data included time of arrival in the hospital, type of bear, types of laceration, wound type, and demographic of patients. Convenience sampling method was used. Point estimate and 95% Confidence Interval were calculated.

**Results:**

Among 2980 patients presenting to the Department of Surgery, the prevalence of bear maul injury was 15 (0.50%) (0.25-0.75, 95% Confidence Interval). Of which, all had laceration injury, with the face and scalp 9 (60%) being the highest injured site. Autumn was the season attacked most in daytime.

**Conclusions:**

The prevalence of bear maul injury was lower than in other studies done in similar settings.

## INTRODUCTION

Bears are fabulous, strong, and agile wild creatures that protect themselves, their young ones, and their territory if they feel in threat.^1^ All bears are impulsive, and dangerous and have the ability to perpetrate severe injuries. Black bears (Ursus thibetanus) are commonly found in the mountainous part of Nepal.^2^

Jumla is situated at an altitude of 2300-6000 m, in the northern part of Nepal which is a suitable layout for black bears' habitat.^3^ Though, they are also reported from other parts of Nepal.^4^ Studies showed that chances of human and bear encounters with injuries to humans were higher in their territory diminished area.^5-7^ However, such encounter-mauling and categorical complex injuries perpetrated by a black bear are scarcely reported from the primary site of the incident happened that lacks the true information.

The aim of the study was to find out the prevalence of bear maul injury among patients presenting to the Department of Surgery in a tertiary care centre.

## METHODS

A descriptive cross-sectional study was done in the Department of Surgery of Karnali Academy of Health Sciences (KAHS) from 1 August 2017 to 1 September 2022 from the medical records. Ethical approval was obtained from the Institutional Review Committee (Reference number: 2078/79/38). All patients who were presented in the Department of Surgery of KAHS with complete hospital data were included in this study. Patients with incomplete hospital data were excluded from the study. Convenience sampling method was used.

The sample size was calculated using the following formula:


$$\begin{array}{ll} \text{n} & ={\text{Z}}^{2}\times \frac{\text{p}\times \text{q}}{{\text{e}}^{\text{2}}}\\ & =1.96^{2}\times \frac{0.50\times 0.50}{0.03^{2}}\\ & =1068 \end{array}$$

Where,

n = minimum required sample size
Z = 1.96 at 95% Confidence Interval (CI)
p = prevalence taken as 50% for maximum sample size calculation
q = 1-p
e = margin of error, 3%

On doubling the sample size, it becomes 2136. However, final sample taken was 2980.

All patients injured by bear maul were instituted broad antibiotics empirically considering all types of bacteria involved. The tetanus injection of 1 ml in buttock (Gluteus) intramuscularly was given. Rabies vaccination was given with the time interval of 0, 3, 7, 14, 28 days. All patients were arrived in hospital within 6 hours and the very same day, thorough debridement, washing of wound with normal saline and proper plastic surgery was given. Those with severe complex facial injuries meticulous surgery was done. Intubation was done to secure the airways. These patients were shifted to Intensive Care Unit (ICU) for few days, later shifted to the general ward for care. The wound was classified on a contamination basis and evaluated by surgeon according to the book "Short Practice of surgery" by Bailey and Love.^8^

Data were collected from the record section of the Department of Surgery. Season, type of bear, sudden or provocative attack, place of incident, and attack time were taken by verbal interview either from patient's relatives or the patient himself after treatment and those recorded data were collected. Patients' records were evaluated with detailed history and injury types from operation theatre (OT) notes and admission files. The author was involved in all cases treated in the hospital.

Data were entered and analysed by using Microsoft Excel 2016. Point estimate and 95% CI were calculated.

## RESULTS

Among 2980 patients presented to the Department of Surgery, the prevalence of bear maul injury was seen in 15 (0.50%) (0.25-0.75, 95% Confidence Interval). The majority of the patients were 30-39 age group with mean age 35.6±10.46 years. Male was dominant 12 (80%) (Table 1).

**Table 1:** Sex and age distribution among patients of bear maul injury (n= 15).

Age	Gender		Number of patients n (%)
	Male n (%)	Female n (%)	
10-19	-	1 (6.67)	1 (6.67)
20-29	2 (13.33)	2 (13.33)	4 (26.67)
30-39	5 (33.33)	-	5 (33.33)
40-49	3 (20)	-	3 (20)
50-59	2 (13.33)	-	2 (13.33)
Total	12 (80)	3 (20)	15 (100)

Among these, contaminated wound 10 (66.67%) and dirty contaminated wound 5 (33.34%) were identified (Table 2).

**Table 2:** Some parameters observed among patients of bear maul injury (n= 15).

Parameters		n (%)
Type of wound	Contaminated wound	10 (66.67)
	Dirty contaminated wound	5 (33.34)
Type of attack	Sudden attack	10 (66.67)
	Provoke attack	5 (33.34)
Time of attack	Morning	2 (13.33)
	Afternoon	13 (86.67)
	Evening	-
Hospital arrival time	Before 3 hours	4 (26.67)
	Within 6 hours	11 (73.33)

Autumn was the season for maximum mauling, happened mostly on day time (Table 3).

**Table 3:** Sociodemographic and seasonal effect on bear maul injury (n= 15).

Parameters	Characteristics	n (%)
Address	Guthichaur (Village)	11 (73.33)
	Others	4 (26.66)
Season	Autumn	13 (86.67)
	Prewinter	2 (13.33)
	Winter	-
	Summer	-

Laceration (simple to complex) wound was common in all cases. The major involved part of the body was the face and scalp 10 (66.67%). However, 2 (13.33%) cases had severe eyeball injury. Out of the total, 1 (6.67%) patient had a femoral vein injury at groin level with a severe hypotensive state (Table 4).

**Table 4:** Types of injury among patients of bear maul injury (n= 15).

Characteristic	n (%)
Scalp and face injury	10 (66.67)
Vascular injury	1 (6.67)
Limb injury	5 (33.34)
Eye injury	3 (20)
Facial bone fracture	2 (13.34)

In spite of devastating injuries, only 2 (13.33%) was referred for second stage of surgery. Mortality was zero.

## DISCUSSION

Bear maul is increasing in current time. However, fewer cases were reported. Majorities of cases may not have reached to the hospital due to geographic, logistic and financial constraints. The prevalence of bear maul in the Department of Surgery of our hospital was found to be 15 (0.50%).

In a study conducted in Kashmir, 417 cases of bear maul injuries were reported in a duration of 17 years whereas in our study 15 cases were reported in a duration of 5 years.^9^ This showed the burden of bear maul injuries in study to be lower than that other study. In our study the number of attack rate was 3 cases per year. Around 40 case are reported worldwide per year which is higher than that of our study.^10^ In another study, only 2 cases of bear attack were reported from Nepal within five years' duration.^7^ If reports of bear maul from other parts of Nepal where bears are observed in abundance were included, the number of bear attack rate would be higher. Thus, by this study we believed that there is a gap in reporting in bear maul which need to be explored and published in totality that may show the prevalence of bear maul number more in Nepal. This needs a proper study collecting data from all over Nepal.

In our study soft-tissue injury occurred in all 15 cases. The facial bone fracture was involved in 2 (13.34%) cases and a large vascular injury in 1 (6.67%) patient. Other parts of the body were less commonly involved, except one in the right thigh with hematoma. Facial fractures in patients included the zygoma, nasal bones and orbital bones dominated. The face and scalp were the most involved injuries 10 (66.67%) in our cases. In other studies, also it is widely agreed that the face and scalp was the most commonly involved part.^9,11-15^ This may be because of the irregular contours of the face and head due to the bony projection that makes these prominent parts easily available for the bear's paws. Subsequently, bears are intelligent animals and they try to weaken their enemies by easily targeting their faces so that the victims are unable to retaliate. In a similar study, it was found that there was usually a delay in hospital reach often due to the difficult place where the incident happens. In study done in central India, in review of 48 patients after bear attack, more than 90% cases reached tertiary centre at 24-48 hours.^16^ In another study done in Kashmir, they reached at centre within 12 hours as all the encounter were in near vicinity from the hospital.^17^ However, all the cases reached in our hospital were within 3-6 hours because all the encountered vicinity was relatively close from our hospital. Those injuries that occurred out of Jumla district were less reported and yet we did not receive a single patient from other district which could be either fatal injuries with mortality on spot or may have taken to another tertiary care centre.

In our study, the tendency of bear inflicted injury seen more in young middle age and attacks were occurred in the autumn season which is comparable to the other study.^11,12,14,20^ The reason for highest attack in those time period is possibly due to descending of bear in the lower land from their habitat for searching food such as maize, apple and other crops as these crops are widely available during this season. Living in villages close to black bear habitats may be another cause of higher chances of encounters with black bear. In our study, black bear mauled patients were young to middle-aged (30-39) and males were predominantly involved 12 (80%) comparable with other studies.^13,20^ This can be explained by the increased outdoor activity by this group. People in this age group, compared with older people, manage work in fields and, at times, go into the forests to collect firewood and grazing cattle. Thirty-three percent of the attacks have been attributed in our study as provocative occurred in village; whereas remaining attack were sudden, encountered either in the jungle or in the village. This result disagrees with other study findings of 6.71% of provocative attack and 92.08% of sudden attack.^10^ Bear entering to the village in search of food where people usually retaliate to bear, may have caused more number of the provocative attack in our study. Most attacks that occurred were at the high grazing land as well as in the village, were during the day agreed with other studies.^1-3^

In the other studies, it was reported, 80% of the injuries were caused by Grizzly bears, 0.9% by a polar bear and 18.1% by an Asiatic black bear.^9,11,17^ However, all of the injuries in our study was reported to be inflicted by an Asiatic black bear because they are the inhabitant of this range of geography.

A study showed that around 71% of injuries among hikers and about 100% of cases among provoked encounters were found to be responsible by sows with cubs.^21^ The attacks caused by the sows with their cubs were less dangerous, thus reflecting that attacks were defensive and triggered by the sow's instinct to protect their cubs. Interestingly in our study, the attack was by Asiatic black bear. No sows with cubs were involved in the attack. In our study, major number of mauls occurred 11 (78.57%) were from the same village which is closed by forest, the habitat of bears, which has a similarity with other studies.^2,5,6^

There were certain limitations of this study. These included the small sample size and single centre with secondary data that would limit its generalisation. Collection of data on bear maul from other parts of Nepal which is above 1500 m in altitude where black bear prevails and analysing on it in totality would give the prevalence of bear maul in Nepal. However, our study at least generated an idea of the prevalence of bear maul injury at the hospital level.

## CONCLUSIONS

The prevalence of bear attack was lower than other studies done in similar settings, with face and scalp being the most common injured site. A wide covered study of bear maul cases including from other part of Nepal is highly recommended to reflect out the exact burden of bear maul cases which will help in planning the safety of people living near to bear's habitat. A quick surgical intervention in earlier presented patients was helpful in majority to decrease complication.
